# Supplementary material for: Development of Films from Spent Coffee Grounds’ Polysaccharides Crosslinked with Calcium Ions and 1,4-Phenylenediboronic Acid: A Comparative Analysis of Film Properties and Biodegradability
Source: Foods. 2023 Jun 28;12(13):2520. doi: 10.3390/foods12132520 (PMC10341353; doi:10.3390/foods12132520)
Supplement: Supplementary file 1 [file foods-12-02520-s001.zip › foods-2405933-supplementary.pdf]

Supplementary Data

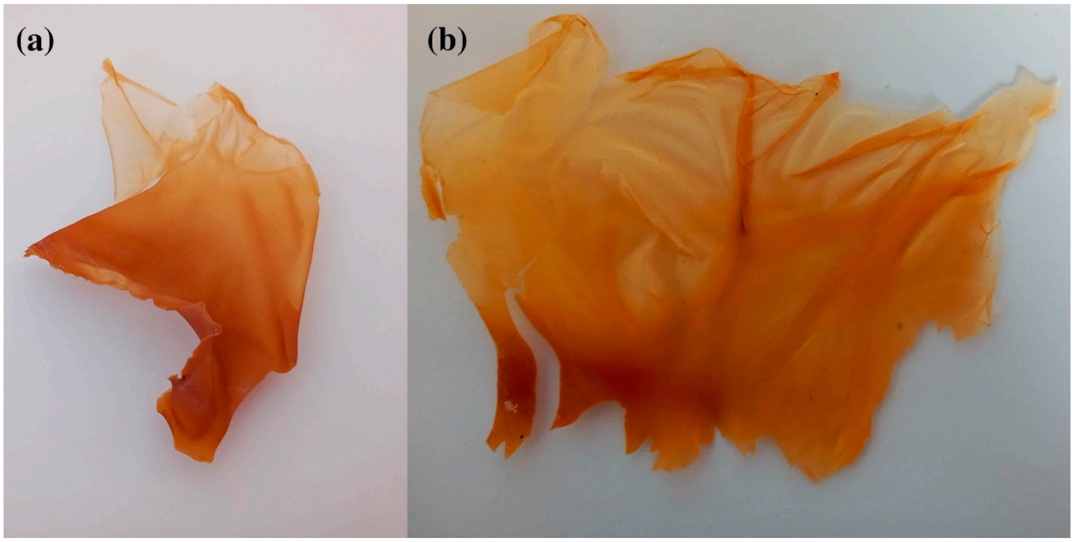

Figure S1. Pictures of samples (a) F311 – after immersion in 2 % $CaCl_2$ solution for 30 min, and (b) F511- after immersion in 2 % $CaCl_2$ solution for 5 min.

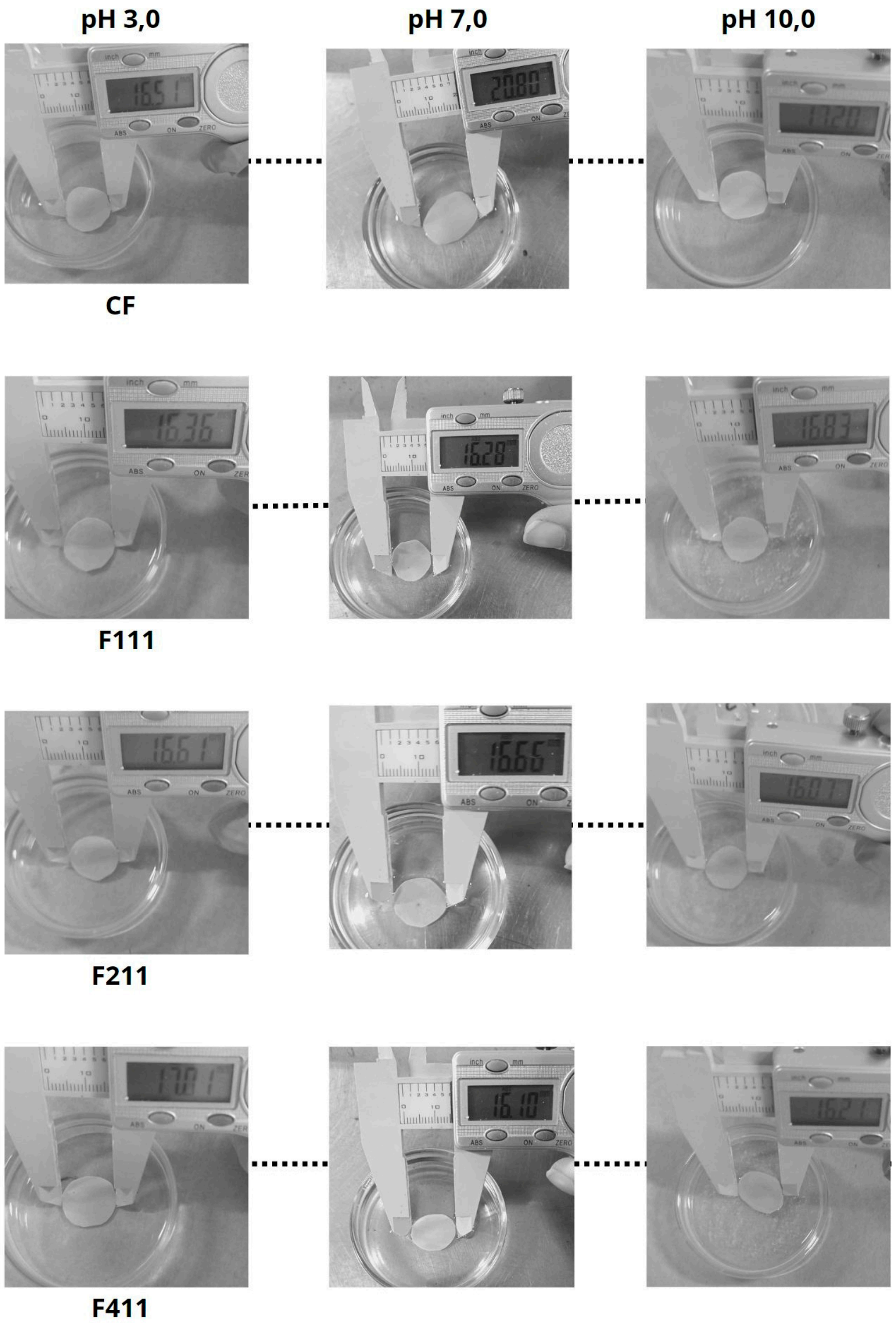

Figure S2. Pictures of the control film (CF) and $CaCl_2$-crosslinked samples (F111 – 6%, 17.5 min; F211 –10%, 5 min; F311 –2%, 30 min; F411 –10%, 30 min; F511- 2% 5 min) immersed in alkaline, neutral and acid medium after 10 days.

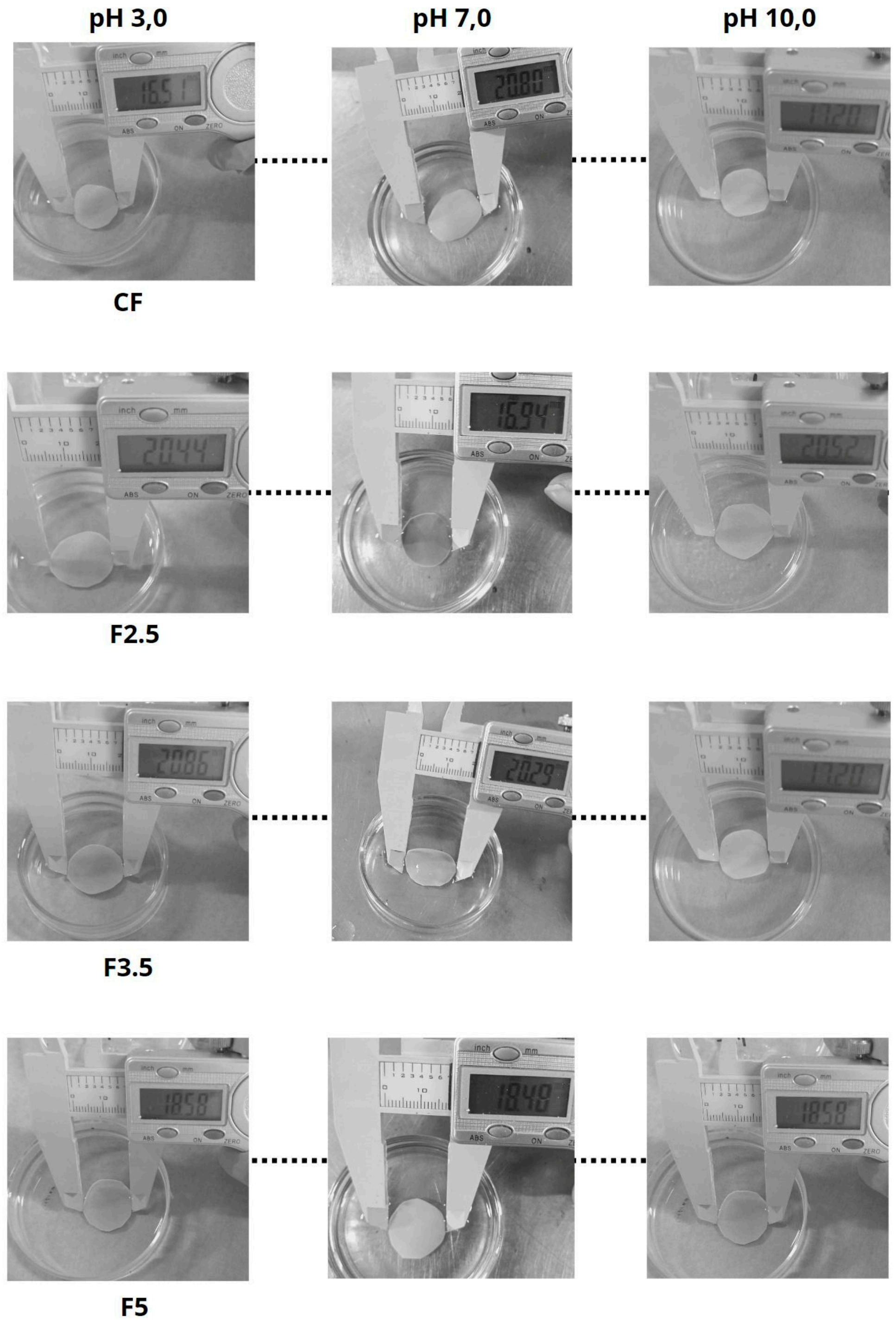

Figure S3. Pictures of the control film (CF) and the samples crosslinked by 1,4-phenylenediboronic acid (PBDA) (F2.5 – 2.5% PDBA; F3.5 – 3.5% PDBA; F5 – 5.0% PDBA) immersed in alkaline, neutral and acid medium after 10 days.

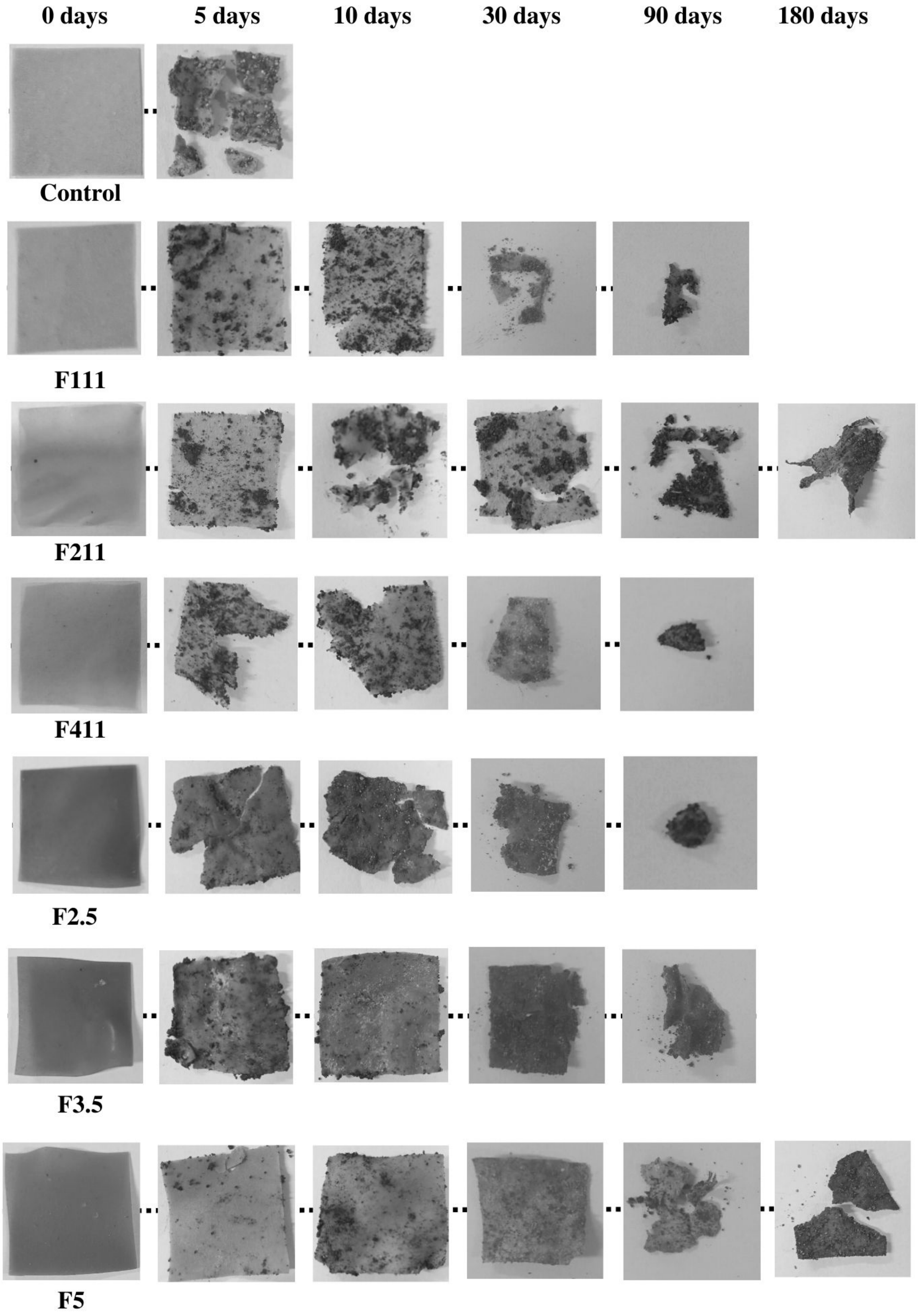

Figure S4: Biodegradability test of the control film, samples immersed in a chloride calcium solution (F111 – 6%, 17.5 min; F211 – 10%, 5 min; F311 –2%, 30 min; F411 – 10%, 30 min; F511- 2% 5 min), and samples with 1,4-phenilenediboronic acid (PDBA) (F2.5 – 2.5% PDBA; F3.5 – 3.5% PDBA; F5 – 5.0% PDBA) as a function of the buried time.
